# Neutrophils: Amoeboid Migration and Swarming Dynamics in Tissues

**DOI:** 10.3389/fcell.2022.871789

**Published:** 2022-04-11

**Authors:** Michael Mihlan, Katharina M. Glaser, Maximilian W. Epple, Tim Lämmermann

**Affiliations:** ^1^ Max Planck Institute of Immunobiology and Epigenetics, Freiburg, Germany; ^2^ International Max Planck Research School for Immunobiology, Epigenetics and Metabolism (IMPRS-IEM), Freiburg, Germany; ^3^ Faculty of Biology, University of Freiburg, Freiburg, Germany

**Keywords:** neutrophil, amoeboid migration, chemoattractants, swarming, inflammation, receptor desensitization, GPCR, infection

## Abstract

Neutrophils are key cells of our innate immune response with essential roles for eliminating bacteria and fungi from tissues. They are also the prototype of an amoeboid migrating leukocyte. As one of the first blood-recruited immune cell types during inflammation and infection, these cells can invade almost any tissue compartment. Once in the tissue, neutrophils undergo rapid shape changes and migrate at speeds higher than most other immune cells. They move in a substrate-independent manner in interstitial spaces and do not follow predetermined tissue paths. Instead, neutrophil navigation is largely shaped by the chemokine and chemoattractant milieu around them. This highlights the decisive role of attractant-sensing G-protein coupled receptors (GPCRs) and downstream molecular pathways for controlling amoeboid neutrophil movement in tissues. A diverse repertoire of cell-surface expressed GPCRs makes neutrophils the perfect sentinel cell type to sense and detect danger-associated signals released from wounds, inflamed interstitium, dying cells, complement factors or directly from tissue-invading microbes. Moreover, neutrophils release attractants themselves, which allows communication and coordination between individual cells of a neutrophil population. GPCR-mediated positive feedback mechanisms were shown to underlie neutrophil swarming, a population response that amplifies the recruitment of amoeboid migrating neutrophils to sites of tissue injury and infection. Here we discuss recent findings and current concepts that counteract excessive neutrophil accumulation and swarm formation. In particular, we will focus on negative feedback control mechanisms that terminate neutrophil swarming to maintain the delicate balance between tissue surveillance, host protection and tissue destruction.

## Introduction

Neutrophils are important effector cells of our immune arsenal and the first line of immune defense for eliminating bacteria and fungi in tissues (Ley et al., 2018; Burn et al., 2021). Large numbers of them circulate in the blood system, constantly prepared to sense signs of tissue inflammation and potential microbe entry. Inflammation-induced activation of blood endothelial cells instructs neutrophils whether and where to leave the blood stream, which involves a well-defined sequence of coordinated interaction events between both cell types (Nourshargh et al., 2010; Margraf et al., 2019). As neutrophils can potentially be recruited to sites of inflammation anywhere in our body and to any organ, they have evolved flexible navigation strategies that allow the infiltration of complex, differentially composed and often dynamically changing tissue compartments.

### Neutrophils—The Prototype of an Amoeboid Migrating Immune Cell

Among immune cells, neutrophils are often considered the prototype of a fast amoeboid-migrating cell. This means that their movement in three-dimensional (3D) and confined environments, including interstitial tissue spaces, does not depend on strong adhesive interactions, allowing an autonomous mode of locomotion independent from the composition of the extracellular tissue environment (Malawista et al., 2002; Lämmermann et al., 2008; Lämmermann et al., 2013). In contrast to mesenchymal cells (e.g. fibroblasts), neutrophils do not require extracellular matrix degradation to move inside tissues. Instead, migrating neutrophils follow paths of least resistance, which can be pores between extracellular matrix fibers or channel-like geometries between anatomical tissue structures or stromal cells (Lämmermann and Germain, 2014). The characteristic protein composition of the neutrophil nucleus, which is donut-shaped in mice and multilobular in humans, adds to the flexibility of mature neutrophils in navigating through dense tissue spaces (Rowat et al., 2013; Wolf et al., 2013; Manley et al., 2018; Salvermoser et al., 2018). Neutrophil amoeboid movement depends on a polarized actomyosin cytoskeleton, with actin polymerization and actomyosin contraction as the two major cellular forces driving migration (Lämmermann and Sixt, 2009). The balanced interplay between branched actin networks at the cell front and myosin II-dependent actin shrinkage at the cell rear allows optimal neutrophil migration. Strikingly, functional interference with one of these components still allows productive, albeit sometimes slower, neutrophil movement in various 3D environments or confined spaces (Lämmermann et al., 2008; Barros-Becker et al., 2017; Fritz-Laylin et al., 2017; Zehrer et al., 2018; Graziano et al., 2019). This highlights the neutrophil as a plastic amoeboid cell, which can use mechanistically distinct migration strategies to move optimally in different microenvironments. Work with human neutrophil-like HL-60 cells on nanopatterned surfaces provided further insight into how coordinated actin assembly supports the low-adhesive mode of 3D neutrophil migration (Sun et al., 2015; Brunetti et al., 2022). Recently, one study showed that the actin regulator Wiskott-Aldrich syndrome protein (WASP) enriches at sites where an external substrate, e.g. a fiber in a fibrillar 3D gel, causes invaginations of the plasma membrane. This facilitates recruitment of the actin-related protein 2/3 (Arp2/3) protein complex and the local assembly of branched actin networks at these sites, which together can exert a pushing force to mechanically create space in dense 3D matrices (Brunetti et al., 2022). Thus, it is not surprising that chemical Arp2/3 blockade substantially lowers the migration speed of randomly moving neutrophils in zebrafish larvae (Barros-Becker et al., 2017), and that neutrophils from patients with a deficiency of ARPC1B, one out of seven subunits of the Arp2/3 complex, show severe migration deficits in 3D matrices (Kempers et al., 2021).

### GPCR Signals—Major Triggers for Neutrophil Polarity and Amoeboid Movement

The induction of an actin-rich neutrophil front and myosin II-rich rear is mostly triggered by inflammatory chemokines or chemoattractants, which act through GPCRs on intracellular signaling pathways to induce cell polarity and locomotion. The exposure to uniform fields of chemokines and chemoattractants is already sufficient to induce neutrophil self-polarization and random movement (Zigmond, 1981). Studies with HL-60 cells identified the fundamental mechanisms of neutrophil self-polarization, revealing crucial roles of PIP3 signaling, small Rho GTPases and regulators of branched actin networks for establishing neutrophil polarity (Xu et al., 2003; Saha et al., 2018; Tsai et al., 2019). It is well established that both soluble and surface-bound attractants support such undirected movement patterns of neutrophil chemokinesis or haptokinesis, respectively. Once neutrophils perceive gradients of soluble or surface-bound attractants, they can also re-orient toward the source of released attractant and undergo directed chemotaxis or haptotaxis (Lämmermann and Kastenmüller, 2019).

**FIGURE 1:**
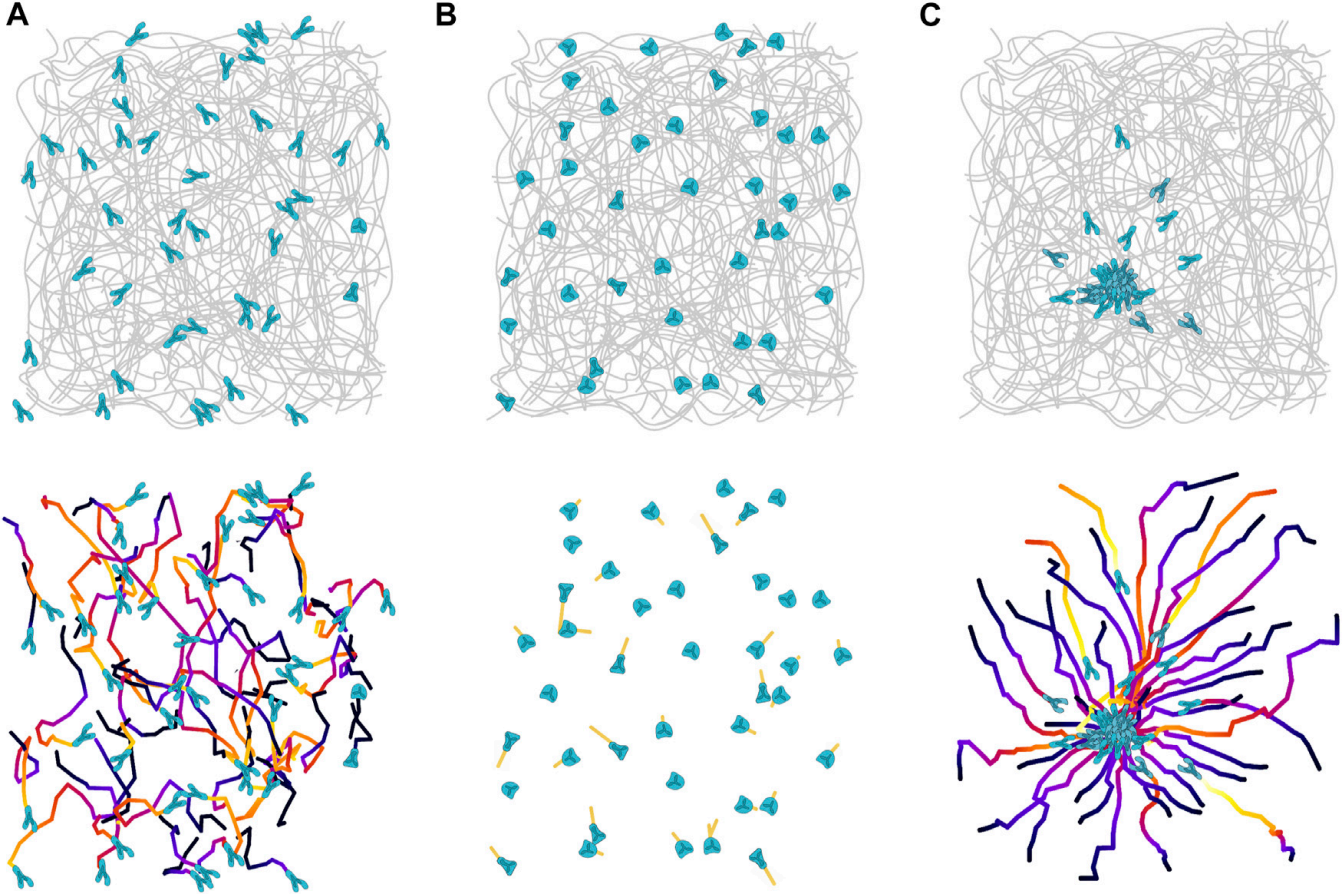
Migration patterns of neutrophils in inflamed skin tissue. **(A)** Amoeboid migrating neutrophils (blue) move rapidly and with polarized morphologies in the interstitial space of diffusely inflamed tissues. The cell trajectories indicate non-directional movement patterns within the collagen bundle scaffold of the dermal interstitium. **(B)** When the inflammatory stimulus dissipates, neutrophils slow down, and eventually lose their polarized morphology and stop migrating. **(C)** However, these neutrophils stay responsive and can still react quickly to acute damages, e.g., a laser-induced tissue injury. Neutrophils in the vicinity of a tissue lesion rapidly polarize and migrate in direction toward the damage site, before a few minutes later swarming is induced and more neutrophils are recruited to form local neutrophil clusters.

Intravital microscopy of neutrophils in inflamed organs reveals migration patterns, which appear non-directional within the interstitial tissue and are seemingly best described as a random walk (Figure 1). However, the underlying processes that lead to these migration patterns are often impossible to determine by just observing the moving cells. Neutrophils migrating at an inflammatory site are exposed to a complex milieu of inflammatory chemokines and chemoattractants, and in most cases we cannot discern if neutrophils perceive attractants as uniform fields or subtle gradients, at low or high concentration. To make our assessment even more difficult, neutrophils of higher vertebrates can respond to more than 30 GPCR signals from a range of chemically diverse chemoattractants, which can all influence the polarization of neutrophil actin and consequently migration in the tissue (Lämmermann and Germain, 2014). On top of that, the specific structure and architecture of a particular tissue compartment adds an additional level of complexity. As the exact geometry and molecular composition of an interstitial space is often unknown, we often cannot assess how physical obstacles shape the trajectories of migrating neutrophils or the distribution of attractant fields. Despite all these problems, we know from several studies that the interstitial migration patterns of low-adhesive neutrophils are largely shaped by the surrounding chemokine and chemoattractant milieu. We have recently reviewed current concepts of GPCR signal crosstalk that guide neutrophil recruitment into and migration within inflamed tissues (Lämmermann and Kastenmüller, 2019). We here focus on one of these concepts, which is particularly relevant for neutrophil amoeboid movement at sites of interstitial tissue injury and infection.

### Neutrophil Swarming—GPCR-Mediated Positive Feedback Amplification to Bring Them all Together

**FIGURE 2:**
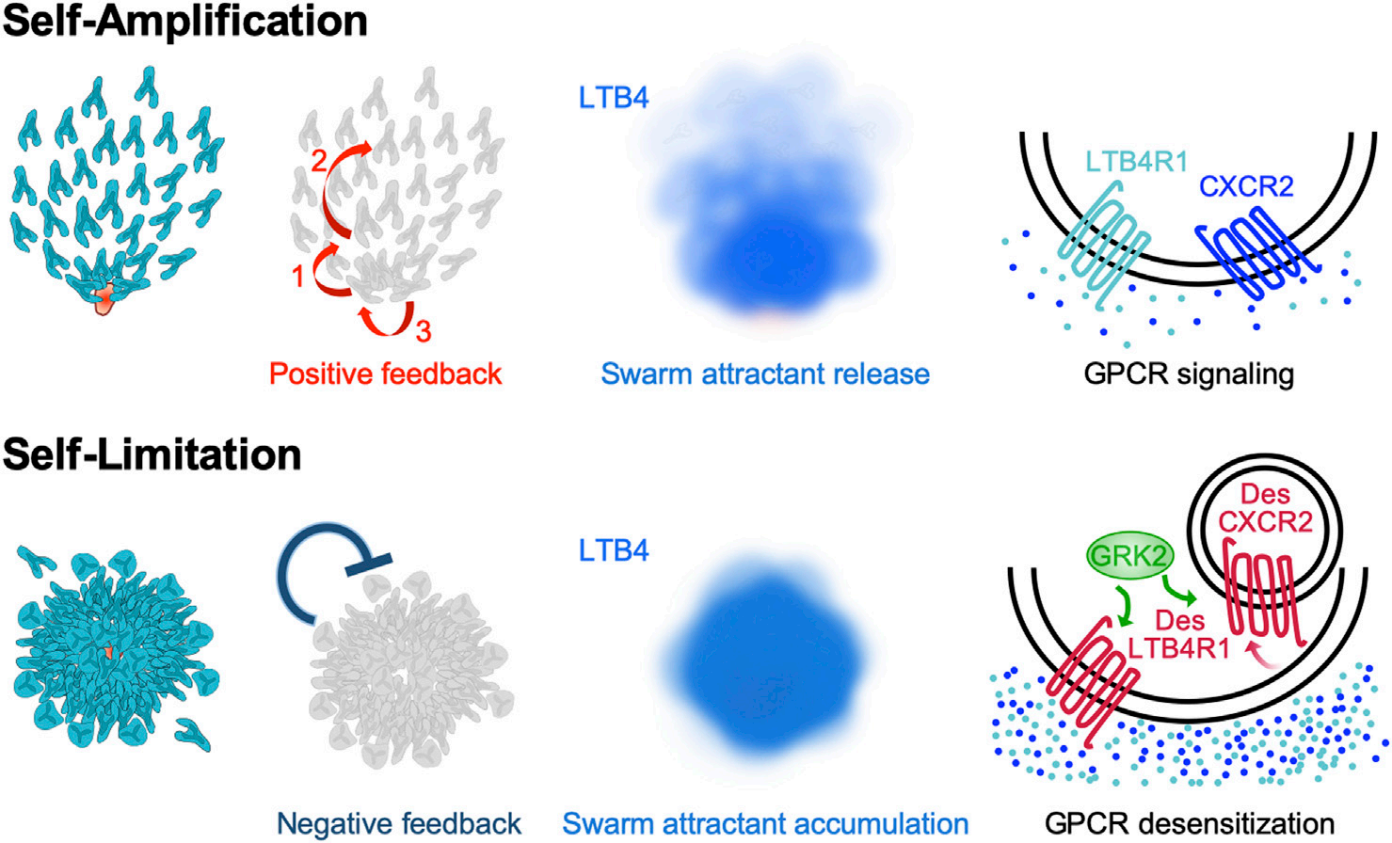
Self-amplification and self-limitation mechanisms that shape self-organized neutrophil swarming behavior. Multiple external signals can trigger neutrophils to release the swarm attractants LTB4 and CXCL2, which self-amplify the formation of neutrophils swarms. Several layers of self-generated signal amplification promote neutrophil swarming and clustering in a feed-forward manner (reviewed in detail in Glaser et al., 2021). This includes (1) a “calcium alarm signal” which propagates in nascent neutrophil clusters, which (2) triggers the release of LTB4 in early-arriving neutrophils. LTB4 acts as a signal relay molecule and acute signal to increase the radius of attraction and recruit more distant cells. Moreover, LTB4 and CXCL2 promote cell aggregation in the developing neutrophil cluster (3). In growing swarm aggregates, the neutrophil-secreted attractants LTB4 and CXCL2 accumulate gradually over time. Neutrophils respond to these high local concentrations of swarm attractants by desensitizing the corresponding swarm attractant receptors LTB4R1 and CXCR2. Desensitization is controlled by the GPCR kinase GRK2 and involves CXCR2 internalization, whereas desensitized LTB4R1 remains at the plasma membrane. Thus, GPCR desensitization serves neutrophils as cell-intrinsic mechanism to self-limit their swarming behavior.

Once neutrophils have entered an inflamed interstitial space, they start exploring the tissue in search of potentially harmful threats, including damaged tissue, acutely dying cells or invaded microbes. Already small tissue lesions or death of few cells can release chemoattractants, which then establish small-sized local gradients in the tissue. Unless resident macrophages manage to “cloak” such micro-lesions and interrupt the formation of an attractant gradient (Uderhardt et al., 2019), neutrophils sense these tissue sites and move directly toward them (Figure 1). Recent work in zebrafish larvae characterized the cytoskeletal requirements for neutrophil reorientation toward such gradients and identified a two-step process: Neutrophils first undergo an exploratory “search” phase, which depends on Arp2/3-mediated dendritic actin networks (Barros-Becker et al., 2017; Georgantzoglou et al., 2021), which is in agreement with HL-60 cell studies in 3D gels (Fritz-Laylin et al., 2017). This is followed by a “run” phase with fast actin flows, cell acceleration and persistence toward the attractant source (Georgantzoglou et al., 2021). Regarding the nature of the released chemoattractants, elegant *in vitro* experiments proposed a concept that pathogen-derived or cell death-associated “end-target” attractants (N-formyl peptides and complement factor 5a (C5a)) can override host tissue-derived “intermediary” attractants (leukotriene B4 (LTB4), chemokine (C-X-C motif) ligand 2 (CXCL2)) to guide neutrophils in a sequential manner from one agonist source to another (Foxman et al., 1997; Heit et al., 2002). Thus, “end-target” attractants can redirect neutrophils out of “intermediary” attractant fields to steer them toward sites of tissue damage and cell death. In addition, low concentrations of “end-target” attractants can stimulate neutrophils to secrete “intermediary” attractants, in particular the lipid LTB4 (Afonso et al., 2012; Sadik et al., 2012), which in turn can relay the initial signal to distant neutrophils that start to follow pioneer cells (Afonso et al., 2012; Lee et al., 2018; Hind et al., 2021). This lipid-mediated amplification mechanism is crucial for the process of neutrophil swarming in which a population of migrating neutrophils switches from random motility to highly directed chemotaxis in order to form local cell clusters under many inflammatory conditions (Lämmermann et al., 2013; Kienle and Lämmermann, 2016) (Figure 2). This population response allows a large number of individual neutrophils to accumulate and concentrate their effector functions at sites of damaged tissue or pathogen invasion.

We have recently highlighted the several layers of positive feedback amplification that underlie neutrophil swarm and cluster formation (Glaser et al., 2021). It is now clear that intercellular communication via the eicosanoid LTB4 is central to the swarming and aggregation response of neutrophils in mouse tissues (Lämmermann et al., 2013), zebrafish larvae (Poplimont et al., 2020; Isles et al., 2021) and *in vitro* setups for human neutrophil swarming (Reategui et al., 2017). LTB4 can be rapidly released by neutrophils within few minutes. Not only “end-target” attractants (N-formyl peptides and complement factors), but also many other molecular triggers (e.g., immune complexes, fungal cell wall components, lipids) are known to induce or boost LTB4 secretion in neutrophils (Kienle and Lämmermann, 2016; Golenkina et al., 2021). All these stimulants have in common that they substantially elevate intracellular calcium levels, which initiates a two-step enzymatic cascade to synthesize and release LTB4 (Luo et al., 2003). Pioneer neutrophils, i.e. the first neutrophils detecting local tissue lesions, were generally viewed as early LTB4 releasers and inducers of the amplified swarming response. Recent work in zebrafish larvae provided more detailed insight into the establishment of the LTB4 gradient in developing swarms (Poplimont et al., 2020). Small groups of clustering pioneer neutrophils in contact with a necrotic wound area generate a “calcium alarm signal”, which propagates across the nascent cell cluster and triggers LTB4 biosynthesis. The propagation of the calcium signal between clustering neutrophils depends on auto- and juxtacrine effects, involving connexin-43 hemichannels, ATP release and ATP-gated calcium channels. These mechanisms together amplify the initial damage signal and promote the local generation of a powerful LTB4 gradient (Poplimont et al., 2020). Besides LTB4, the chemokine CXCL2 acts as another swarm attractant of mouse and human neutrophils, and appears to function synergistically with lipid signaling (Lämmermann et al., 2013; Reategui et al., 2017; Kienle et al., 2021). It is currently assumed that CXCL2, which can also be released by mouse neutrophils (Li et al., 2016; Girbl et al., 2018), exerts a more local effect at the site of neutrophil clustering, as its binding capacity to glycosaminoglycans likely prevents diffusion into the tissue (Rajasekaran et al., 2012). Thus, neutrophils secrete swarm attractants to self-amplify swarming and aggregation in a feed-forward manner, which provides neutrophils a level of self-organization in complex inflammatory tissue environments (Figure 2).

### Neutrophil Swarming—GPCR-Mediated Negative Feedback Control to Stop Excessive Swarm Formation

How this swarm attractant-driven amplification is stopped and uncontrolled neutrophil accumulation prevented has long remained unclear. Recently, we identified a critical role of GPCR desensitization for stopping the swarming behavior of mouse neutrophils (Kienle et al., 2021). Homologous desensitization of one GPCR type as a response to high concentrations of its ligand is a well-known phenomenon for almost all neutrophil-expressed GPCRs. It differs from heterologous desensitization, where one GPCR signal can act as desensitizing signal for multiple other GPCR types, causing immune cells to become refractory to several chemotactic stimuli. Based on many *in vitro* studies, it is well established that human and mouse neutrophils undergo homologous GPCR desensitization and become unresponsive to repeated or continuous agonist stimulation (Lämmermann and Kastenmüller, 2019). *In vitro* studies had further shown that the threshold levels of agonist-induced GPCR desensitization in neutrophils can be ∼10-fold or higher than those needed for maximal calcium flux and chemotaxis (Rose et al., 2004). Based on such studies, GPCR desensitization was discussed as a potential terminal stop signal when neutrophils reach the center of an inflammation where chemoattractant concentrations are the highest (Rose et al., 2004). However, direct *in vivo* proof for this concept was missing because of technical limitations in measuring the exact local concentrations of endogenous attractants in living tissues. As we are lacking critical information on whether and where migrating neutrophils sense “high” attractant concentrations *in vivo*, the role of GPCR desensitization for neutrophil navigation in inflamed tissues and host defense has remained largely unresolved. Based on our previous findings that neutrophils release LTB4 and CXCL2 (Lämmermann et al., 2013), we reasoned that neutrophils self-generate fields of highly concentrated attractants at sites where cells accumulate in larger numbers. We hypothesized that a local temporal increase of swarm attractants might cause the desensitization of the respective GPCRs for LTB4 (LTB4R1) and CXCL2 (CXCR2). To functionally interfere with GPCR desensitization, we targeted GPCR kinases (GRKs), which are critical serine/threonine protein kinases that can prevent the overstimulation of cells through excessive GPCR stimulation (Freedman and Lefkowitz, 1996). Following GPCR ligand binding, GRKs can phosphorylate the intracellular C-termini of GPCRs, creating high-affinity binding sites for the recruitment of β-arrestins. As a consequence, the increased binding of β-arrestin to the receptor sterically hinders the interaction of GPCR and G proteins. This uncoupling of G proteins from the receptor prevents further GPCR signaling in a situation of repeated agonist stimulation. Additionally, β-arrestin binding also targets the receptors for internalization, and sometimes degradation (Lämmermann and Kastenmüller, 2019). Neutrophils have been reported to express four GRK isoforms (GRK2, GRK3, GRK5, and GRK6) and previous studies with neutrophils had started to functionally connect them to GPCRs for “intermediary” attractants: GRK2 to CXCR1 (Raghuwanshi et al., 2012) and CXCR2 (Fan and Malik, 2003; Alves-Filho et al., 2010; Lee et al., 2015), GRK5 to CXCR2 (Fan and Malik, 2003), GRK6 to LTB4R1 (Kavelaars et al., 2003) and CXCR2 (Raghuwanshi et al., 2012). Studies on GRK-mediated control of “end-target” attractant binding N-formyl peptide receptors (FPRs) remained partly contradicting (Liu et al., 2012; Subramanian et al., 2018). Given the uncertainties from published literature, we decided to systematically identify the GRK isoforms that are functionally relevant for swarming. We studied primary mouse neutrophils that were depleted of individual GRKs and also neutrophils that were lacking all four GRK isoforms (Kienle et al., 2021), and tested their migratory response toward gradients of the primary swarm-mediating attractants LTB4 and CXCL2. Our experiments identified GRK2 as the key GRK isoform to control the desensitization of LTB4R1 and CXCR2, with only minor functional impact on other neutrophil-expressed GPCRs, including FPRs and C5a receptors (Kienle et al., 2021). Two-photon intravital imaging of injured skin and infected lymph nodes showed that GRK2-controlled GPCR desensitization is crucial for neutrophil swarming in mouse tissue. GRK2-depleted neutrophils, which were desensitization-resistant to swarm attractants, failed to stop their migration at sites where swarming neutrophils accumulate. Moreover, GRK2 knockout neutrophils moved faster and explored larger areas of tissues infected with the bacterium *Pseudomonas aeruginosa*. However, mice with such fast and desensitization-deficient neutrophils showed impaired rather than enhanced bacterial clearance. Our study revealed that defective GRK2-controlled arrest during neutrophil swarming comes at the cost of suboptimal phagocytosis and containment of bacteria (Kienle et al., 2021). Moreover, it highlighted that GPCR desensitization acts as a neutrophil-intrinsic negative feedback control mechanism to self-limit neurophil swarming (Figure 2). Thus, desensitization to a self-produced amplification signal is an important mechanism underlying the self-organization of neutrophil swarms (Rocha-Gregg and Huttenlocher, 2021b; Kienle et al., 2021). Many novel questions arise from this finding, as, for example, the role of GPCR reactivation after desensitization and how this regulation affects neutrophil swarm phenotypes.

An important role of GPCR desensitization for neutrophil swarming has also been shown in zebrafish larvae at sites of tissue wounding (Coombs et al., 2019). Zebrafish CXCR1, which is functionally considered very related to mouse CXCR2, promotes neutrophil recruitment and the formation of neutrophil clusters (Powell et al., 2017; Coombs et al., 2019). At sites of neutrophil aggregation this receptor becomes rapidly desensitized and internalized (Coombs et al., 2019). Zebrafish CXCR1 desensitization is critical to allow dispersal of neutrophils away from the wound site. This transition involves a switch to signaling through zebrafish CXCR2, which does not become desensitized, remains at the plasma membrane and steers neutrophils away from cell clusters (Coombs et al., 2019). Movement away from a focal accumulation of neutrophils is often referred to as “reverse migration”, a phenomenon that is well studied in the zebrafish model (Nourshargh et al., 2016; Robertson and Huttenlocher, 2022). Such neutrophil behavior commonly requires a two-GPCR system (Lämmermann and Kastenmüller, 2019), which allows neutrophils to lower their response to retention signals at the site of cell clustering and increase their response to signals in the immediate tissue surrounding (Hind and Huttenlocher, 2018; Rocha-Gregg and Huttenlocher, 2021a). Neutrophil migration out of cell clusters and re-routing to other clusters can be often observed under conditions of transient swarming in infected mouse tissues (Chtanova et al., 2008; Kienle and Lämmermann, 2016; Kienle et al., 2021). Mouse neutrophils, which clustered together and became desensitized by swarm attractants, remain responsive to new tissue insults. This is in agreement with the before discussed concepts that “end-target” attractants can override “intermediary” swarm attractants, which allows the redirection of neutrophils from cell aggregates to novel sites of cell death in the inflamed or infected tissue.

GPCR desensitization to swarm attractants likely synergizes with other mechanisms to stop neutrophil swarming. As an example, it has been shown for swarming human neutrophils that they start producing lipids involved in the resolution of inflammation, including lipoxin A4 and resolvin E3 (Reategui et al., 2017). These lipids may act as potential stop signals by antagonizing LTB4-mediated signaling in neutrophils. However, direct proof that this mechanism acts in inflamed tissues is missing. In addition, the potential roles of tissue-derived signals, which may antagonize pro-migratory LTB4 signaling (Arita et al., 2007; Mizuno et al., 2014), remain to be investigated in future studies of neutrophil swarming.

### Reactive Oxygen Species, NADPH Oxidase and Neutrophil Extracellular Traps—Control Mechanisms for Neutrophil Swarming?

To eliminate invaded microbes from our tissues, neutrophils use several effector mechanisms, including phagocytosis, the release of antimicrobial products into the extracellular space (degranulation), and the formation of neutrophil extracellular traps (NETs) (Rosales, 2018; Burn et al., 2021). Reactive oxygen species (ROS) generated by the multisubunit enzyme NADPH oxidase are involved in most of these neutrophil antimicrobial defense strategies. ROS can act intracellularly in the phagosome, activate the release of granules, induce NET formation and be itself released into the extracellular tissue space (Nguyen et al., 2017). Whether and how neutrophil migration and ROS generation are functionally linked at sites of inflammation and infection has been of long-standing interest. ROS sensing by the transient receptor potential melastatin-related 2 (TRPM2) cation channel has been demonstrated to restrain neutrophil migration (Wang et al., 2016). *Trpm2*-deficient neutrophils show enhanced directed migration responses to several chemokines and chemoattractants, in particular N-formyl peptides as classical “end-target“ attractants. It was suggested that chemoattractant-stimulated neutrophils generate ROS that can be sensed by TRPM2 through oxidation on its N-terminal Cys549 residue. Interaction of the oxidatively modified TRPM2 with the N-formyl peptide receptor FPR1 induces FPR1 internalization and desensitization, which arrests neutrophils in attractant gradients. An involvement of GRK2 in this process was discussed (Wang et al., 2016). However, whether calcium-permeable TRPM2 acts similarly on the swarm-mediating receptors LTB4R1 and CXCR2, respectively, was not addressed. A recent study with neutrophils from chronic granulomatous disease (CGD) patients, who suffer a primary immunodeficiency resulting from inactivating mutations in NADPH oxidase, revealed novel insight into neutrophil calcium homeostasis and LTB4 production (Song et al., 2020). Surprisingly, CGD neutrophils produce higher amounts of LTB4 after activation with the fungal wall component zymosan or immune complexes, compared to control cells. The absence of NADPH oxidase leads to increased calcium influx and an overload of calcium in neutrophils, which amplifies LTB4 production. In consequence, mutant neutrophils form numerous and larger neutrophil clusters in the presence of zymosan *in vitro* (Song et al., 2020) or in response to micropatterned fungi (Hopke et al., 2020). Thus, NADPH oxidase limits LTB4 production by acting on calcium entry in neutrophils, explaining how increased production of LTB4 can exacerbate neutrophil recruitment and inflammation in CGD.

ROS generated from NADPH oxidase and myeloperoxidase are also required for NET release. In response to many external triggers, e.g. microbial stimuli, neutrophils release nuclear contents into the extracellular space to trap and kill microbes, a process known as NET release. The formation of NETs involves the mobilization of granule contents, the processing of histones, the disintegration of intracellular membranes and the rupture of the neutrophil cell membrane (Sollberger et al., 2018). As a result of this process, a mixture of nucleoplasm and cytoplasm is expelled to form NETs, which are extracellular depositions of decondensed chromatin decorated with granule-derived proteins, such as active proteases and anti-microbial peptides. When neutrophils undergo cell death as a consequence of this whole process, it is referred to as NETosis (Yipp and Kubes, 2013; Boeltz et al., 2019). While NETs are best known as crucial antimicrobial defense strategy, it is now also established that NETs play important roles in sterile inflammation, autoimmunity and other pathophysiological conditions (Boeltz et al., 2019). Thus, it is not surprising that the influence of neutrophil death and NET formation on neutrophil swarming behavior has been of recent interest and under debate over the last years. To make a long story short, the formation of neutrophil swarms can occur in the absence of dying neutrophils. As an example, human neutrophils, which induce LTB4 secretion in response to micropatterned heat-killed *Staphylococcus aureus* particles or zymosan, undergo robust swarming responses without any signs of cell death (Kienle and Lämmermann, 2016; Reategui et al., 2017). However, neutrophil fragments indicative of cell death have often been observed by intravital microscopy in the center of neutrophil swarms of mice and zebrafish (Chtanova et al., 2008; Peters et al., 2008; Yipp et al., 2012; Lämmermann et al., 2013; Kamenyeva et al., 2015; Uderhardt et al., 2019; Poplimont et al., 2020; Isles et al., 2021). It is currently assumed that lytic cell deaths, including pyroptosis, necroptosis and necrosis, occurring at the site of neutrophil aggregation influence the swarming response, likely by the additional release of intracellular neutrophil-attracting molecules (Kienle and Lämmermann, 2016). When NET formation is associated with cell death, neutrophils undergo a suicidal lytic form of NETosis, which could potentially also release attractants. A recent study in zebrafish showed that the inhibition of NET release mediators (gasdermins, neutrophil elastase, myeloid-specific peroxidase) decreases neutrophil swarm frequency (Isles et al., 2021). However, it remained unresolved how and when during the swarming process NET release may exert the swarm-promoting effect. Although early-recruited neutrophils at sites of local wounding had been observed to release chromatin in form of large balloon-like structures in the zebrafish model, a clear correlation between this form of lytic cell death and neutrophil swarm initiation could not be established (Poplimont et al., 2020; Isles et al., 2021). Instead, early-recruited neutrophils that flux calcium as a potential readout for LTB4 synthesis serve mostly as center of attraction for following neutrophils (Poplimont et al., 2020). When human neutrophils form swarms in response to micropatterned pathogenic fungi, NETs could not be observed during the initial swarm recruitment. However, NET release at later stages of neutrophil swarming restricts and delays fungal growth through the action of NADPH oxidase and myeloperoxidase (Hopke et al., 2020). The long-term effects of NET release for neutrophil swarm termination have not yet been explored. Previous studies have shown that aggregated NETs can disrupt neutrophil recruitment by degrading cytokines and chemokines under certain inflammatory conditions (Schauer et al., 2014; Hahn et al., 2019). Thus, it might be worth exploring whether NETs play a role for the maintenance of swarm attractant gradients in large neutrophil aggregates. Together, our current knowledge on the roles of ROS, NET release, NETosis and other forms of cell death for neutrophil swarming is still limited and needs to be explored in more detail in the future.

## Conclusion

Amoeboid neutrophils are perfectly adapted to rapidly move toward almost any site in the interstitial space, where microbial invaders may cause harm to the organism. This flexibility is supported by their cell deformation-driven, low-adhesive mode of migration, which makes neutrophils largely autonomous from any tissue-specific environment. To optimize the search for damaged tissue and elimination of pathogens, the individual neutrophils can coordinate their dynamics and act as a swarming cell population. This allows neutrophils to combine the individual effector responses of many cells, focalize them and prevent the spreading of any harmful threat in the tissue. GPCR-driven positive feedback amplification provides neutrophil swarming robustness and a level of self-organization, which is required to overcome the complex attractant milieu of most inflammatory tissue settings. Self-organized neutrophil swarming also relies on GPCR desensitization, which acts as negative feedback control mechanism to prevent excessive neutrophil swarming and maintains the balance between tissue surveillance, host protection and tissue destruction. How neutrophil swarming dynamics and effector functions are functionally linked and spatiotemporally controlled in groups of clustering neutrophils remains to be fully investigated. This interplay will be of future interest, as many external triggers, e.g. microbial products and “end-target” attractants, induce both ROS generation and LTB4 signaling by raising intracellular calcium. Tissue-derived signals likely influence all of these aspects of self-organized swarming and future work will uncover how tissue factors influence and shape the neutrophil swarm phenotype in different organs and tissue compartments.
